# High fat diet is protective against kidney injury in hypertensive-diabetic mice, but leads to liver injury

**DOI:** 10.1371/journal.pone.0281123

**Published:** 2023-02-02

**Authors:** Véronique Cheff, Mayra Trentin-Sonoda, Amélie Blais, Jean-François Thibodeau, Chet E. Holterman, Alex Gutsol, Christopher R. J. Kennedy, Richard L. Hébert

**Affiliations:** 1 Department of Cellular and Molecular Medicine, University of Ottawa, Ottawa, Ontario, Canada; 2 Kidney Research Centre, Division of Nephrology, Department of Medicine, Ottawa Hospital Research Institute, Ottawa, Ontario, Canada; 3 Liminal BioSciences Inc., Laval, Québec, Canada; Auburn University, UNITED STATES

## Abstract

Chronic kidney disease (CKD) is a worldwide health burden with increases risk of end-stage renal function if left untreated. CKD induced in the context of metabolic syndrome (MS) increases risks of hypertension, hyperglycemia, excess body fat and dyslipidemia. To test if combining a high-fat diet (HFD) regimen onto the hypertensive/ diabetic phenotype would mimic features of MS induced-CKD in mice, hyperglycemia was induced in genetically hypertensive mice (Lin), followed by HFD regimen. For that, 8-week-old male were subjected to streptozotocin (STZ) intraperitoneal (i.p.) injections (50 mg/kg, 5 days consecutive). LinSTZ were fed a 60% kCal HFD for 8 weeks. Lin mice treated with STZ developed polydipsia, became hypertensive and hyperglycemic. HFD induced weight gain, protected against glomerular hypertrophy, scarring, and albuminuria at endpoint compared to regular diet fed LinSTZ. On the other hand, HFD induced steatosis, liver fibrosis, inflammation, and increase in AST/ALT ratio, characteristics of non-alcoholic liver disease. Taken together, our results show that LinSTZ mice fed a HFD did not lead to a more robust model of MS-induced CKD, protected against kidney injury, but inducing liver damage. More studies are necessary to understand the kidney protective mechanisms of HFD when superimposed with hypertension and type 1 diabetes.

## Introduction

Metabolic syndrome (MS) is a multifactorial disease, characterized by the simultaneous presence of cardiovascular disease (CV), hyperglycemia, diabetes, and dyslipidemia [1, 2].

Hypertension is closely related with the development of chronic kidney disease and to end stage renal disease (ESRD) [3, 4]. Metabolic syndrome increases the risk of developing cardiovascular diseases [5, 6] and mortality rate associated to cardiovascular complications [6].

The worldwide prevalence of diabetes mellitus (DM) is a major concern in the modern society [7]. Diabetic kidney disease (DKD) is a multifactorial disease, characterized by fibrotic lesions in the kidney, persistent inflammation, oxidative stress and renal microvascular injury. Hyperglycemia also promotes dyslipidemia and obesity via the accumulation of fat in adipocytes, and obesity by itself is a risk factor for MS inducing diabetes. Moreover, adoption of unhealthy lifestyles can lead to obesity, thus increasing the risk for DKD [8].

Accumulation of fat can also occur in other tissues, and if accumulated in the liver, it can lead to fatty liver disease, characterized by the accumulation of lipids in hepatocytes. Chronic liver disease is a risk factor contributing to non-alcoholic fatty liver disease (NAFLD). Liver injury inducing NAFLD increases the risk of CKD by twofold [9, 10]. Taken together, hypertension, hyperglycemia and obesity are MS major factors contributing to renal injury if left untreated and leading to CKD progression.

The current mouse models of advanced-CKD do not fully mimic human renal disease and cellular mechanisms dysfunctions. In the current study, we first aimed to obtain a novel mouse model of metabolic syndrome induced CKD mimicking human disease with hyperglycemia and dyslipidemia phenotypes combination in the LinA3+ mouse. We also aimed to investigate whether MS induced via hypertension, hyperglycemia and dyslipidemia could lead to liver injury. We found that LinSTZ mice had elevated systolic blood pressure, reverted by high fat diet (HFD) regimen. Hypertensive-diabetic mice also displayed significant kidney injury, and HFD partially protect against kidney function impairment and fibrosis. Furthermore, HFD induced liver injury, inflammation, and fibrosis. Our results indicate that high fat diet can be protective in the CKD progression, but it plays a detrimental role to the progression of liver diseases.

## Methods

### In vivo study

In this study, we used hypertensive TTRhRen (or also named LinA3+), previously characterized in the literature [11, 12]. Briefly, liver-specific expression of a modified human pro-renin cDNA transgene was achieved under the control of a 3-Kb region of the mouse transthyretin promoter.

Briefly, from 8 to 10 weeks-old LinA3+ and wildtype (WT) littermate male mice on a FVB/n background were subjected to 5 days of intraperitoneal (i.p.) injections of STZ (50mg kg^-1^BW^-1^; Sigma-Aldrich, Oakville, ON.) or 0.1 M Na-citrate buffer pH 4.5 as vehicles respectively to induce hyperglycemia via pancreatic beta cell death [13–15]. After 4 weeks post-STZ injection, mice were allocated in corresponding groups and fed either a regular diet (10% kilocaloric (kCal); Teklad, Mississauga, ON.) or a high-fat diet (HFD; 60% kCal; Teklad, Mississauga, ON.) for 8 weeks for body weight gain [16, 17].

All mice were subjected to baseline and endpoint metabolic cages. Food consumption, water intake and urine output were measured at 24 hours metabolic cages for baseline and 12 hours for endpoint. Data obtained from the 12 hours metabolic cages were extrapolated to obtain 24 hours values comparisons.

Distress was monitored throughout the study and mice were euthanized before they reached the humane endpoint. Mice displaying signs of dehydration in the hypertension/ hyperglycemic groups were supplemented with i.p. saline solution and with heat pad for up to 8 hours at the time. At the endpoint, mice were anesthetized with 5% isoflurane and euthanasia occurred by exsanguination (cardiac puncture); blood and tissue were collected and further allocated for different assays.

Experimental animals (male, 8–20 weeks) were housed at the Animal Care Facility at the University of Ottawa with free access to food and water. Ethical approval was obtained from the University of Ottawa Animal Care Committee (under protocol number CMM-2209) and the study was conducted according to the guidelines of the Canadian Council on Animal Care.

### Blood pressure measurement

Systolic, diastolic and pulse reading were measured by tail-cuff plethysmography (BP 2000, Visitech systems, Apex, NC) throughout the study, as previously described [18]. Mice were trained for 5 consecutive days at 11 weeks of age (5 preliminary readings, 10 actual readings/ day). Later BP measurement in the study was obtained within 2 consecutive days on 12- and 20-weeks old mice. False results from unread blood pressure instrument and outliers measured according to the average +/- two-times standard deviation of sample were removed from data analysis to obtained sample average.

### Physiological data

Baseline blood samples were collected by saphenous vein bleed into heparinized capillaries (Fisher Scientific, Pittsburgh PA). The capillaries were centrifuged 5000 g for 10 minutes at 4°C to measure hematocrit fraction before sampling plasma and place immediately -80°C until subsequent analysis. Sacrifice blood samples were collected via cardiac puncture into heparinized syringes, kept on ice and centrifuged at 5000 g for 10 minutes at 4°C. Collected plasma was immediately frozen at -80°C until subsequent analysis. Plasma glucose levels were determined by glucometry (Nova StatStrip Xpress Glucose CR Meter. Nova Biomedical, Waltham, MA).

At sacrifice, kidneys were removed, individually weighted and normalized to tibia length. The right kidneys were cut sagittal. A section of the right kidney was placed in 4% paraformaldehyde fixation solution for 24 hours, transferred to 70% ethanol before embedded in paraffin. The other sagittal right kidney sections were placed in 30% sucrose for 24 hours and embedded in the OCT frozen section compound. The left kidneys were snap-frozen for protein and quantitative PCR (qPCR) analysis.

### Biochemistry analysis

Plasma and urine biochemistry values were measured according to Cres17 (sodium, chloride, total protein, albumin, globulin, creatinine, glucose, cholesterol, triglycerides) and Cres18 (urine sodium, urine chloride, urine total protein, urine urea nitrogen, urine creatinine, urine glucose) respectively test type from IDEXX inc. (IDEXX, Westbrook, Maine). The values of the urine obtained for biochemistry were normalized for the 24 hours urine output (obtained by metabolic cages, as described before).

### Albuminuria

Albuminuria was measured using the Mouse Albumin Elisa Kit (Bethyl labs, Montgomery, TX.) following manufacturer’s protocol and measured A_450_ nm wavelength spectrophotometry (FLUOstar Galaxy. BMG LABTECH, Cary, NC). Albumin levels were determined by normalizing to creatinine concentration, determined by high pressure liquid chromatography (HPLC) creatinine area under the curve (AUC) concentration quantification by fluorescence detection and with the polar bonded-phase 300-SCX ZORBAX narrow-bone 5-micron column (Agilent Technologies 1100 series, Santa Clara, CA) with 15 mM sodium acetate, 40% methanol and 10% acetonitrile (pH 4.2) for creatinine migration into column [19].

### Insulin test

Baseline plasma insulin level was measured using Ultra-Sensitive Mouse insulin Elisa kit (Crystal Chem, Elk Grove Village, IL) following manufacturer’s protocol. Plate was measured using the spectrophotometry (FLUOstar Galaxy. BMG LABTECH, Cary, NC). Insulin concentration was measured by subtracting A_630_ to A_450_ nm and interpolated using the standard curve.

### Histology and α-SMA immunohistochemistry

PFA Paraffin-embedded kidney sections (3 μm), liver (4μm) and WAT (4 μm) were obtained and stained with periodic-acid Schiff (PAS) or Masson’s Trichrome reagent [18]. All sectioning, paraffin embedding, and staining were performed by the University of Ottawa’s Department of Pathology. The sections were viewed using a light microscope at either 200X or 400X magnification (Axioskop 2 Imager A1, Zeiss, Germany). Kidney glomerular (20–25 glomeruli/ mice) were analyzed using imaging software (Axiovision v4.8, Carl Zeiss, Germany) was used to calculate relative mesangial matrix/ glomerular area, whereby the area of the mesangial scar as a percentage of total glomerular area was determined. Visual degree of kidney and liver damage was scored using the following qualitative scale: 0—no damage in tissue, 1—minimal, 2—mild, 3—moderated, 4—marked and 5—severed damage and injury. A total of (20–30) representative visual areas were analyzed in a blinded manner for each group.

Kidney and liver α-smooth muscle actin (α-SMA; Santa Cruz Biotechnology, Dallas, TX.) immunofluorescence was performed on paraffin-embedded sections mounted on glass slides. Sections were deparaffinized in mixed xylenes (Fisher Scientific, Pittsburgh, PA.), and rehydrated through a gradient of ethanol and distilled water. Sections were washed 3x in PBS, boiled for 20 minutes in 0.1 M Na-citrate buffer (pH 9.0) for antigen unmasking. Sections were blocked in PBS containing 10% donkey serum/ 1% BSA for 1 hour and incubated with mouse anti-α-smooth muscle actin (1:200) overnight at 4°C. Slides were washed and treated with a FITC-labelled donkey anti-mouse secondary antibody (1:1000; Molecular Probes, Burlington, ON.) for 1 hour, followed by 4,6-diamidino-2-phenylindole (DAPI; Sigma-Aldrich, Oakville, ON.) for nuclear localization. Sections were covered with fluorescent mounting medium (Vector laboratories, Burlington, ON.) and coverslips. Immunofluorescence sections were visualized under fluorescence microscopy at 400X magnification, whereby representative cortical profiles from each group were obtained in a blinded manner.

### Quantitative real-time RT-PCR

Quantification of the human pro-renin liver specific transgene superimpose into the LinSTZ mice model and SGLT-2, tumor necrosis factor alpha (TNF-α) expressions was assayed to the wildtype (WT) littermates. Briefly, snap-freeze liver and kidney tissues collected at sacrifice from each group and RNA were isolated using QIAGen RNEasy Minikit with DNase treatment kit manufacturer’s kit protocols (QIAGen, Toronto, ON.). Quantification of isolated RNA was performed using the Epoch Spectrophotometer with Take3 Micro-Volume Plate (BioTek, Winooski, VT.) as described by the manufacturer. RNA analysis was undergone using Gen5 software (Version 2.09) (BioTek, Winooski, VT.) calibrated for RNA quantification. Extracted RNA was converted to cDNA using the High-Capacity cDNA Reverse Transcription kit (Applied Biosystems, Foster City, CA.) with 500 ng starting material per reaction before performing Real-Time RT-PCR. Reactions were carried using SYBR Advantage qPCR Premix (Clontech Laboratories, CA, USA) on an ABI Prism 7000 Fast Sequence Detection System (Applied Biosystem) and with corresponding pairs of primers found in **S1 Table**. Briefly, human renin (hRen) and mouse renin (mRen) primers were normalized to control mouse gene GAPDH, and the mouse SGLT-2 and TNF-alpha genes was normalized to the 18S control gene. The comparative threshold cycle method was used for data analyses. All samples were assayed in duplicate. Relative expressions were analyzed to the 2^-ΔΔ^CT qPCR threshold. A relative quantification was performed for each receptor and results are expressed as fold change control.

### ALT and AST Elisa assay

Liver function was measured using the alanine aminotransferase (ALT) and liver damage was measured using aspartate aminotransferase (AST) according to the Elisa kits and following the manufacturer’s protocol (MyBioSource inc., San Diego, CA. and Abcam inc. Toronto, ON. respectively) [on endpoint plasma samples from each group].

### Angiotensin II Elisa assay

Angiotensin II in plasma samples was measured using the Angiotensin II Elisa Kit (Enzo, Cedarlane distributor, Burlington, ON.) following manufacturer’s protocol and measured A_450_ nm wavelength spectrophotometry (FLUOstar Galaxy. BMG LABTECH, Cary, NC).

### Statistics

GraphPad Prism (Version 7.0a) was used to present the data and perform statistical analysis (Graphpad Prism, San Diego, Ca.). Values are expressed as means 土 standard error of the mean (SEM). Statistical analysis comparing two groups was done using unpaired t-test with parametric Gaussian distribution test. Multiple groups analysis was statistically analyzed by two-way ANOVA and multiple comparison done by with Tukey post-test. A p-value < 0.05 with n ≥ 3 was considered statistically significant.

## Results

### Characterization of LinSTZ / HFD model

As part of the model characterization, mice were placed in metabolic cages for at least 12h. Data collected from the metabolic cages showed that LinSTZ mice displayed symptoms of polydipsia, characterized by a higher water intake and urine production, compared to WT mice, whereas HFD partially reduced those parameters. The increase in hematocrit fraction (red blood cells) was also observed in the LinSTZ group (Table 1). Further signs of dehydration were observed when analysing urine osmolarity, since LinSTZ had significant lower values compared when compared to non-hypertensive and non-diabetic WT littermate (Table 1). Hypertensive diabetic mice had a decrease in body weight compared to WT (Table 1).

**Table 1:** Effects of HFD on polydipsia, hematocrit, and body weight gain.

	WT	WT HFD	LinSTZ	LinSTZ HFD	P value
**Water intake (m/daul)**	2.9 ± 0.1	2.5 ± 0.7	22.6 ± 0.6*^&^	13.2 ± 4.0*^&#^	*p <0.001 vs WT,
					^&^p<0.05 vs WT HFD,
	n = 16	n = 8	n = 31	n = 22	^#^p<0.01 vs LinSTZ
**Urine volume (ml)**	0.4 ± 0.1,	0.2 ± 0.1	19.0 ± 0.8*^&^	9.8 ± 0.7*^&#^	*p<0.0001 vs WT,
					^&^p<0.0001 vs WT HFD,
	n = 16	n = 8	n = 31	n = 22	^#^p<0.0001 vs LinSTZ
**Urine osmolarity**	3675.0 ± 389.2	4580.0 ± 832.1	1139.0 ± 13.4*^&^	1337.0 ± 40.1*^&^	*p<0.0001 vs WT,
	n = 15	n = 8	n = 31	n = 23	^&^p<0.0001 vs WT HFD
**HCT (%)**	48.9 ± 0.5,	48.7 ± 1.9	54.6 ± 0.6*^&^	49.5 ± 1.9^#^	*p<0.01 vs WT,
					^&^p<0.05 vs WT HFD,
	n = 16	n = 8	n = 32	n = 22	^#^p<0.01 vs LinSTZ
**Body weight (g)**	27.13 ± 0.31	135.60 ± 9.4*	25.44 ± 0.28^&^	121.40 ± 7.0*^#^	*p<0.0001 vs WT,
					^&^p<0.0001 vs WT HFD,
	n = 15	n = 8	n = 32	n = 23	^#^P<0.0001 vs LinSTZ

No significant differences were observed in the hematocrit fractions compared to WT fed regular diet (Table 1). However, HCT was lower in LinSTZ HFD compared to LinSTZ (Table 1). HFD regimen led to significant increases in body weight in both WT and LinSTZ mice (Table 1), compared to mice fed a regular diet. LinSTZ mice fed a HFD were able to gain significantly more weight when compared to LinSTZ fed a regular diet, validating obesity on the model.

### STZ induces diabetes type 1 in genetic hypertensive mice

In order to generate a model that mimics metabolic syndrome-induced kidney injury, we opted for subjecting hypertensive/ T1DM mice (LinSTZ) to subjecting obesity/dyslipidemia induced by high fat diet. To this end, wildtype (WT) or LinSTZ mice were fed either a regular or HFD and LinA3+ mice were rendered hyperglycemic via a low-dose streptozotocin challenge (50 mg/kg BW i.p. for 5 consecutive days) [20].

**Fig 1:**
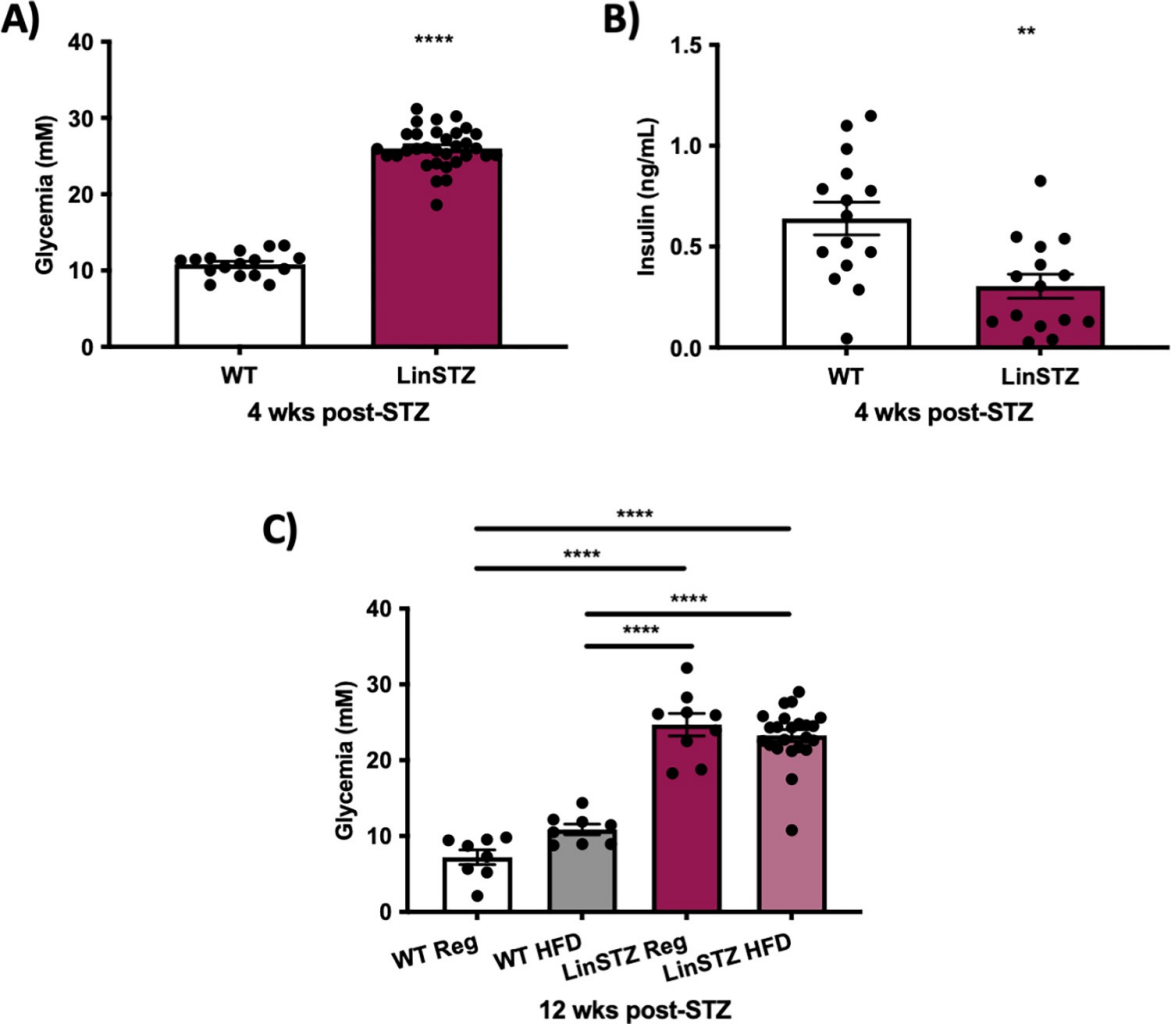
Hyperglycemia and hypoinsulinemia is observed in LinSTZ mice. A) Baseline fasted blood glucose is higher in LinSTZ. B) Plasma insulin is decreased in LinSTZ mice at 12 weeks of age. C) Endpoint blood glucose remained elevated in LinSTZ mice. The high blood glucose was induced in fasted mice through five consecutive daily low-dose STZ intraperitoneal injections (50 mg/kg BW/ day). Blood glucose results were obtained using glucometer via saphenous vein from 4 hours fasted mice. Data expressed as mean 土 SEM, A and B) unpaired t test, ****p<0.0001 **p<0.01, C) ****p<0.0001.

An increase in fasting blood glucose level was observed in the LinSTZ group compared to their WT littermates receiving only sodium citrate buffer as vehicle (Fig 1A). Accordingly, STZ led to reduced fasting insulin levels in LinSTZ mice, confirming beta cells injury-mediated hyperglycemia (Fig 1B). Blood glucose levels remained elevated and unaffected in both regular and HFD fed LinSTZ mice at endpoint (Fig 1C).

### HFD does not further increase LDL/VDL ratio

**Fig 2:**
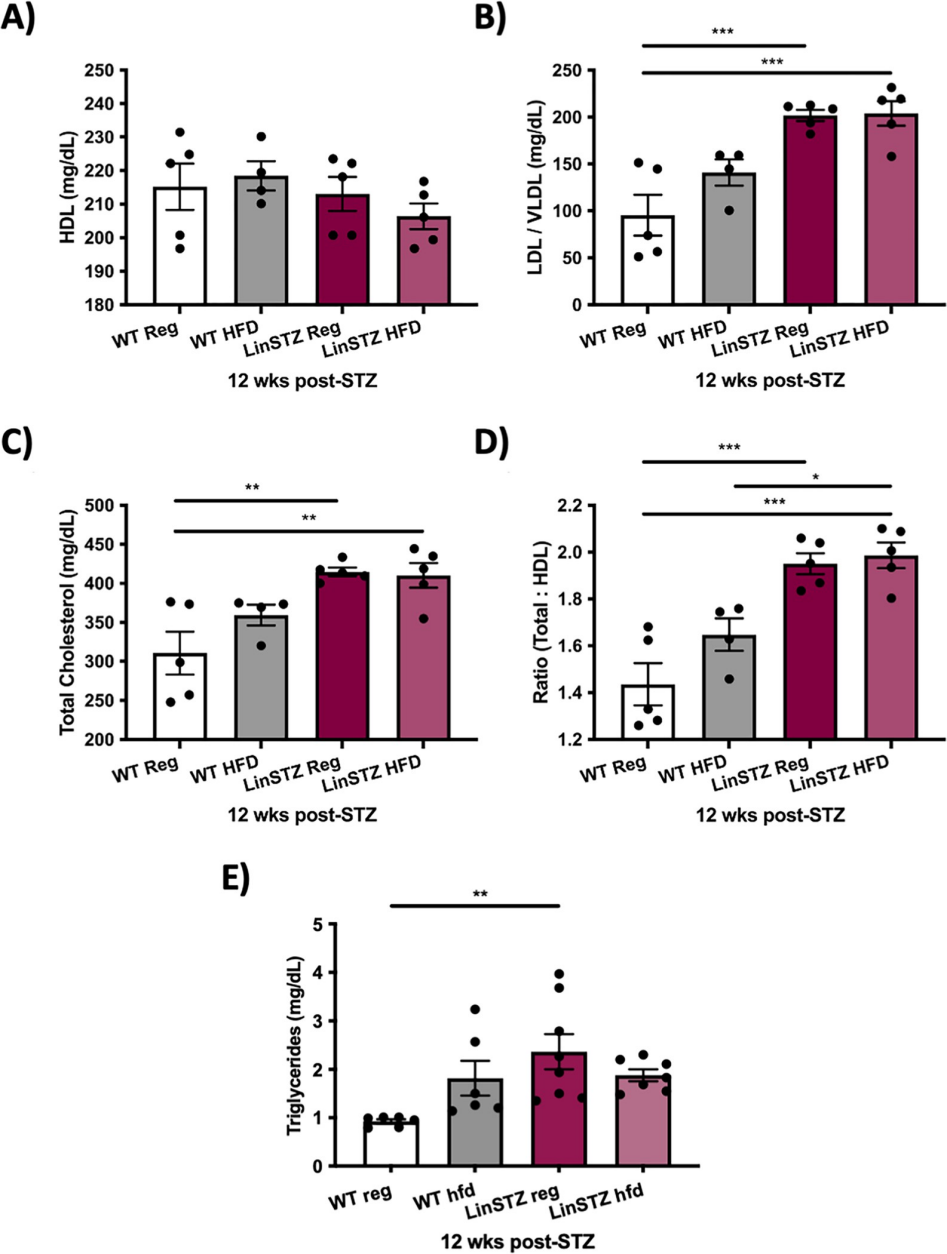
Plasma triglyceride and cholesterol LDL/VLDL are increased in LinSTZ mice. A) Quantification for high density lipoprotein (HDL) from plasma samples. B) Low density and very-low density lipoprotein (LDL/ VLDL) quantification. C) Total cholesterol concentration. D) Cholesterol ratio obtained by dividing the total cholesterols by the HDL values. E) Free triglycerides in plasma. Data expressed as mean 土 SEM, *p<0.05, **p<0.01 or ***p<0.001.

To verify if diabetes and high fat diet would have an impact in circulating levels of cholesterol, high-density lipoprotein (HDL) and low-density lipoprotein (LDL) were analyses in plasma samples. HFD had no impact in HDL (Fig 2A). Plasma LDL cholesterol was elevated in LinSTZ mice, confirming dyslipidemia (P<0.001, Fig 2B), but it was not aggravated by treatment with HFD (Fig 2B, as well as total cholesterol (Fig 2C). Cholesterol ratio in mice was determined by dividing the total cholesterol by HDL values. LinSTZ mice a had higher cholesterol ratio compared to their WT littermates (P<0.001, Fig 2D). Circulating triglyceride was increased in the LinSTZ group (Fig 2E).

### High fat diet has no significant impact on blood pressure Lin mice

**Fig 3:**
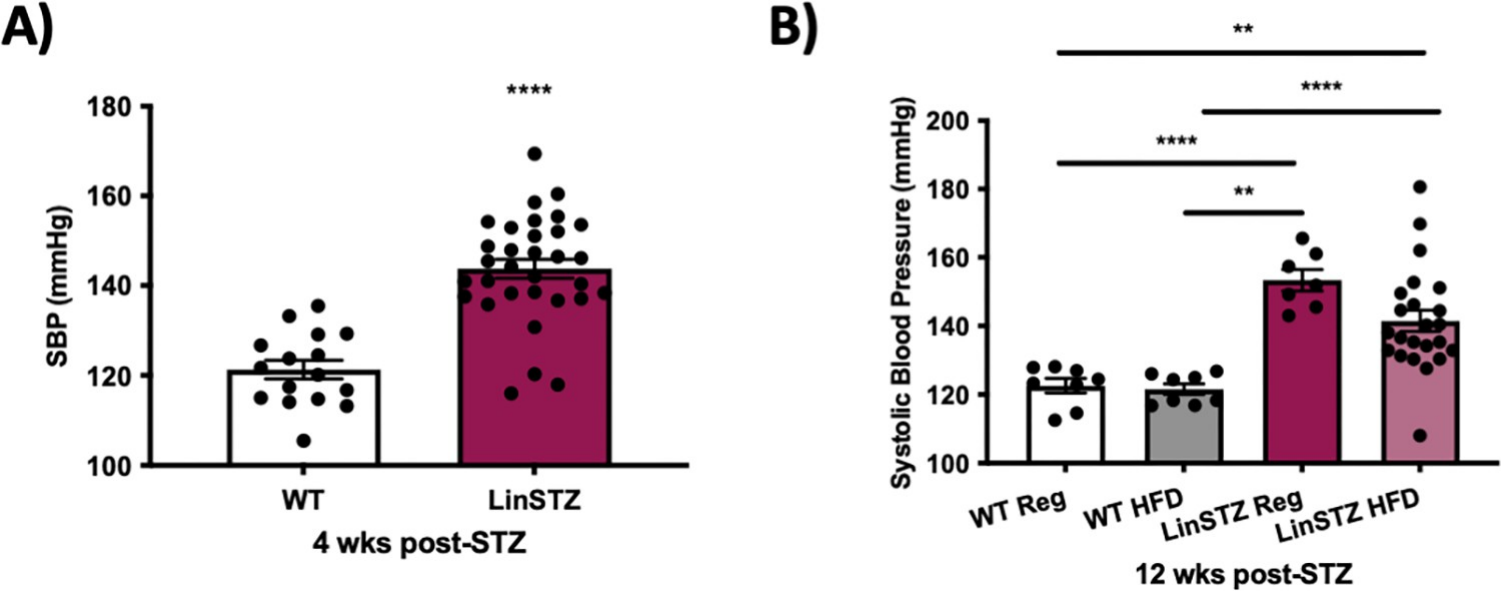
Systolic blood pressure taken from WT and LinSTZ mice given a 60% kCal high fat diet (HFD). A) High systolic blood pressure was confirmed at baseline. B) LinSTZ mice fed regular diet sustain increase in SBP at 12 weeks post STZ and HFD shows a trend improving systolic blood pressure in LinSTZ after 8 weeks. Results represent mice aged from 12 (baseline) to 20 (endpoint) weeks of age. Data expressed as mean 土 SEM, A) unpaired t test, ****p<0.0001 B) ANOVA, **p< 0.01, ***p = 0.001, and ****p<0.0001.

LinA3+ mice are hypertensive at birth, due to sustained increased renin-angiotensin system activity [18]. Using tail-cuff plethysmography-based systolic blood pressure measurements, we confirmed that systolic blood pressure (SBP) was higher in genetically hypertensive LinSTZ at baseline, compared to WT littermates (P<0.0001, Fig 3A). Moreover, the SBP remained elevated in LinSTZ mice fed a regular diet throughout the study. HFD had no significant effect on SBP in LinSTZ mice, although a trend to decrease was observed (p = 0.10, Fig 3B).

### HFD protects against kidney injury

**Fig 4:**
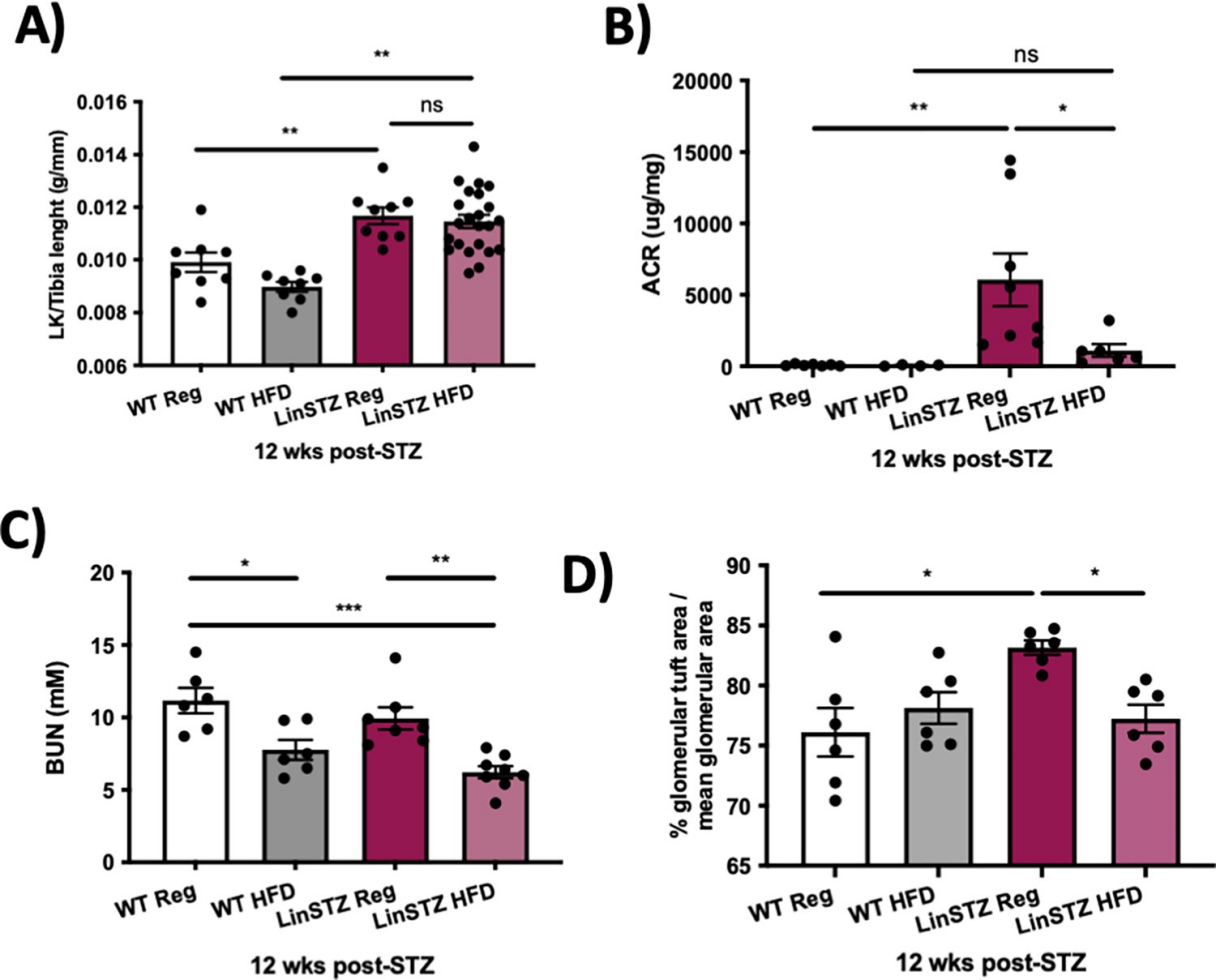
LinSTZ mice have higher urinary albumin to creatinine ratio (ACR) and ameliorated BUN in presence of HFD. A) Left kidney weight to tibia length ratio. B) LinSTZ mice show a trend towards decreasing ACR after 8 weeks of HFD. C) Blood urea nitrogen illustrating kidney function amelioration with HFD. D) Glomerular tuft area to total glomerular are ratio. Data expressed as mean 土 SEM. *p<0.05, **p<0.01 or ***p<0.001, and ns: not significant.

To investigate if high fat diet combined with hypertension and diabetes would lead to CKD, we performed kidney morphometrical/morphological and functional evaluations. LinSTZ mice displayed increase in left kidney weight to tibia length ratio (Fig 4A). HFD did not protect against kidney hypertrophy (Fig 4A). Kidney injury was also analyzed by measuring the albumin-to-creatinine ratio (ACR) at baseline (pre-HFD regimen) and endpoint (4 weeks post-HFD regimen). LinSTZ mice were already albuminuric at baseline with confirmed hypertension, and hyperglycemic phenotypes (Fig 4B). At the endpoint, LinSTZ mice on fed HFD for a duration of 8 weeks were significantly less albuminuric compared to regular diet LinSTZ mice (Fig 4B). The addition of a HFD regimen resulted in significant BUN reduction in both WT and LinSTZ mice (Fig 4C). Additionally, LinSTZ shows an increase in glomeruli tuft, reverted by HFD regimen (Fig 4D).

**Fig 5:**
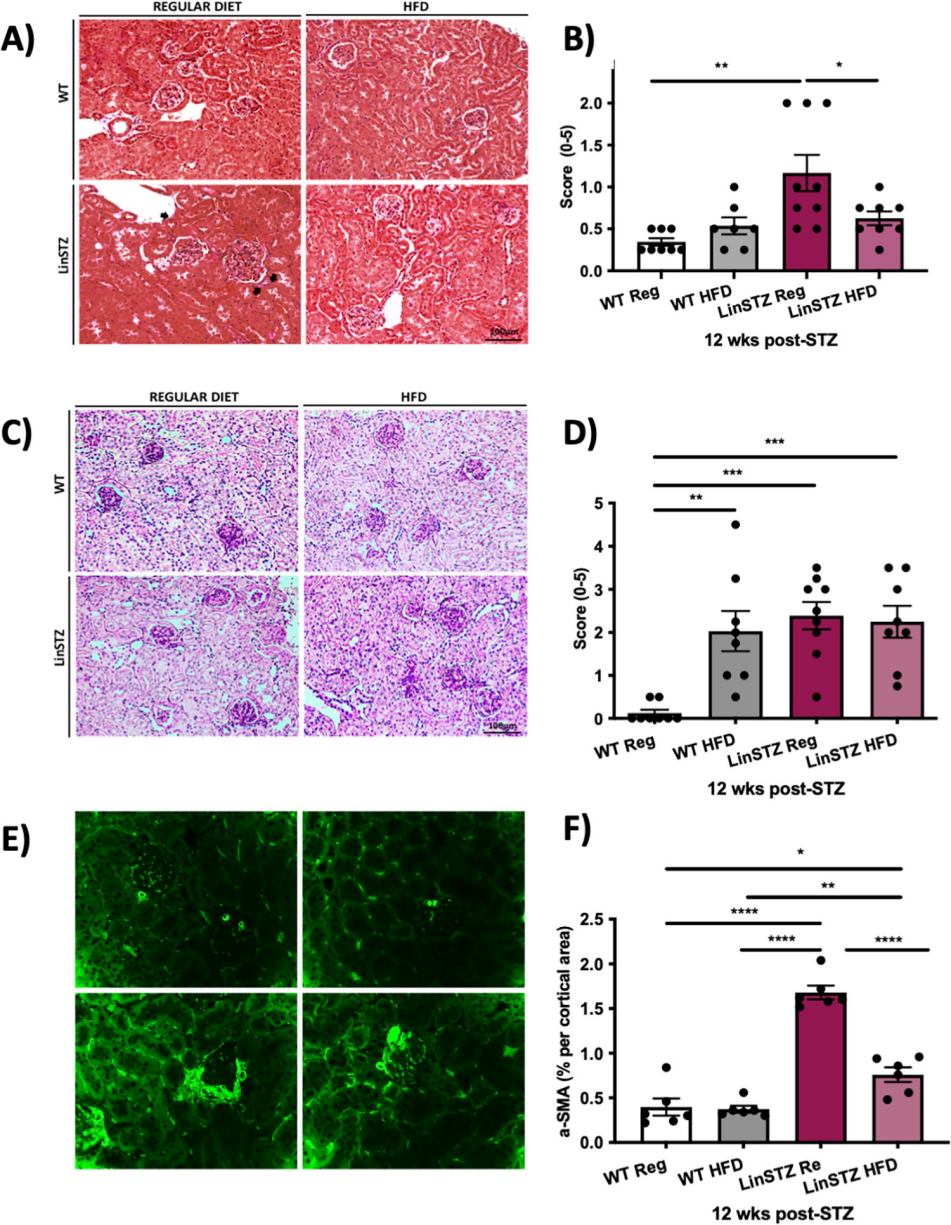
LinSTZ mice significantly increased tubulointerstitial injury. A) Visual representation of collagen deposition by Masson’s trichrome staining. Magnification = 200X. B) Masson’s trichrome scoring of kidney section revealing increase of collagen deposition in the glomeruli and tubulointerstitial of LinSTZ mice fed a regular diet. C) Representative image of PAS staining. D) injury score analysis based on PAS staining. E) Representative images of Immunostaining for αSMA. F) quantification for αSMA staining. Data expressed as mean 土 SEM, *p<0.05, **p<0.01, ***p<0.0001, and ****P<0.00001. Scale bar = 100um.

Histological assessment of kidney injury was performed on paraffin-embedded kidney sections stained with Masson’s trichrome staining. The evidence of collagen proliferation and deposition, fibrotic glomerulopathy (CG) and tubulointerstitial fibrosis (TIF) quantified by Masson’s trichrome was observed in the LinSTZ vehicle group (Fig 5A). HFD-fed LinSTZ mice showed less renal injury as Masson’s trichrome staining intensity by its reduction back to basal level, (Fig 5B). Injury score was analyzed by PAS staining, showing that HFD diet alone induces increase in injury, but it does not further aggravate injury in LinSTZ mice (Fig 5C and 5D).

Alpha SMA has shown to be a relevant marker of reduced renal function as it can be identified in the renal glomerular mesangial cells and interstitial myofibroblasts. Kidney sections were stained for alpha smooth muscle actin (αSMA). There was a significant increase in αSMA in the LinSTZ mice, but the expression is reduced when mice are on HFD (Fig 5E and 5F).

### HFD induces liver injury, preserving renin expression in LinSTZ mice

**Fig 6:**
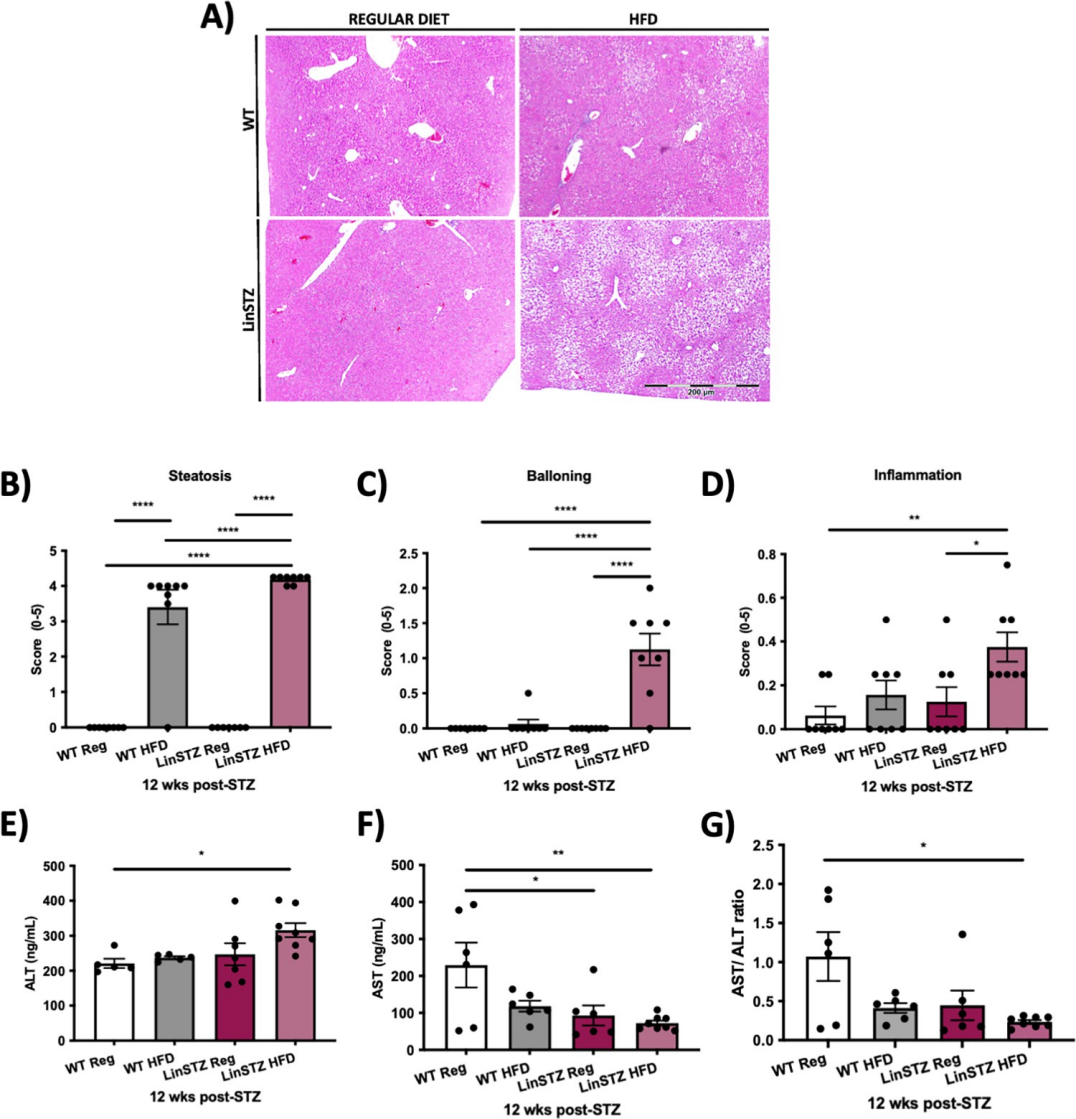
Liver injury analysis. A) PAS staining of liver samples, pictures taken at magnification 400x. B) Steatosis score based on PAS staining. C) Ballooning score based on PAS staining. D) Inflammation Score based on PAS staining. E) Alanine aminotransferase is reduced in LinSTZ mice on Regular and HFD and WT HFD illustrating a decrease in liver function. F) Aspartate aminotransferase increased in LinSTZ HFD mouse model illustrating liver damage G) ALT to AST ratio. Data expressed as means 土 SEM, *p<0.05, **p<0.01, ***p<0.0001, and ****P<0.00001.

Liver damage was assessed by PAS staining to identify inflammation, fibrosis and apoptosis. Morphological changes in LinSTZ mice treated with HFD can be observed in Fig 6A. The histological assessment of liver injury revealed that both WT HFD and LinSTZ HFD mice developed steatosis (fat accumulation) lesions (Fig 6B) when compared to regular diet fed mice. In fact, we observed a 4-fold increase in steatosis when mice were fed HFD, compared to regular diet fed mice. LinSTZ HFD steatosis was significantly higher than WT HFD (Fig 6B). The liver histological scoring analysis revealed pronounced hepatocyte ballooning (Fig 6C) and inflammation (Fig 6D) in HFD-fed LinSTZ mice. There was a significant increase in ALT in LinSTZ mice compared to the WT group (Fig 6E), and a significant decrease in AST for LinSTZ mice fed with HFD compared to WT fed regular diet (Fig 6F). The ratio AST/ALT was significantly decreased for LinSTZ HFD mice when compared to WT fed regular diet (Fig 6G).

The humanized pro-renin transgene is specifically expressed in liver hepatocytes of LinA3+ mice. We questioned whether renoprotection seen in HFD-fed LinSTZ mice was due to HFD impact in the liver, and subsequently affecting the severity of the LinSTZ phenotype. To validate this hypothesis, we customized oligo pair primers to the human renin sequence and performed a quantitative PCR (S1 Table). As expected, WT littermate’s level of detection is undetectable for human renin expression by qPCR since they do not express the transgene (S1A Fig). Moreover, the human renin mRNA level of expression was not affected by the degree of liver damage, as a 250- and 300-fold change is observed in LinSTZ regular and HFD-fed mice respectively (S1A Fig).

As we observed a drop in hypertension in the LinSTZ HFD mouse model, we also quantified Angiotensin II (Ang II) as a potential target to validate the RAAS system function. By enzyme-linked immunosorbent assay (Elisa), we quantified Ang II from endpoint plasma collected in LinSTZ mice fed a regular and HFD. Again, the Ang II plasma level was unaffected in the model (S1B Fig).

## Discussion

Each component of metabolic syndrome can be associated with worsening of kidney function and injury, leading to chronic kidney injury [17, 21–23]. In this study we attempted to stablish a model of metabolic syndrome inducing-CKD that recapitulates human disease and therefore better study disease progression. First, we observed that HFD had no impact in blood glucose or cholesterol levels. Second, we showed that genetically hypertensive mice with diabetes type 1 induced by STZ has elevated systolic blood pressure. Third, HFD induces increase in body weight, but seems to revert the increase in blood pressure. Fourth, LinSTZ mice have advanced kidney injury and impairment in function, that was reverted by HFD regimen. And last, HFD induced liver injury.

In our model, we utilized a superimposition of the genetically induced hypertension, type-1 diabetes (LinSTZ), and a high fat regimen within a timeframe of 20 weeks. The LinSTZ mouse showed features which are similar to the MS criteria. LinSTZ and LinSTZ HFD had multiple signs of dehydration and polydipsia (Table 1), as illustrated in the current literature [24, 25]. HFD had a significant weight gain, resulting in apparent obesity (Table 1), characteristic of a HFD regimen [16, 17].

STZ induced promoted a hyperglycemic state, mimicking a T1DM phenotype [14, 26]. Although it has been previously demonstrated that HFD can reduce blood glucose levels, inducing glycemic control in STZ-induced type 1 diabetes [27], we showed that glycemic levels were preserved in LinSTZ HFD (Fig 1) when compared to hypertensive diabetic mice.

Several mouse models of human progression of metabolic syndrome have been proposed to date [1]. However, studies present in the literature have demonstrated that mice were able to achieve MS phenotypes but fail to fully mimic human disease progression by not fully achieving levels of kidney failure. In fact, an estimated 24 to 30 weeks is required to generate renal diseases in a mouse model [2, 28]. LinSTZ mice had higher levels of circulating cholesterol, when compared to WT group (Fig 2), while HFD did not aggravated increase in cholesterol levels (Fig 2). Hyperglycemia leads to *de novo* lipogenesis, explaining the increase of cholesterol observed in the LinSTZ group [29, 30]. In the early stages of non-alcoholic fatty liver disease, there is an increase in lipid export, but it has been shown to plateau or even decrease with progression of the disease [31]. We hypothesize that HFD-induced liver injury could explain the lack of changes in circulating levels of cholesterol.

As expected, genetically modified Lin mice became hypertensive, and high blood pressure was sustained after induction of diabetes type 1 (Fig 3). HFD attenuated increase in SBP in LinSTZ mice (Fig 2), ACR, glomerular injury and in the tubulointerstitial fibrosis (Figs 4 and 5). These contradict some studies in the literature, demonstrating renal damage progression in HFD-fed mouse models [32–34]. However, a recent study performed by Pour Abassi et al. 2022 showed that low-carbohydrate high fat diet can offer cardiovascular protection in obese adults [35], indicating that further studies are necessary to understand the mechanisms behind high fat diet regimen in CVDs.

LinSTZ mice showed impairment in kidney function, showed by ACR and BUN measurement (Fig 4) and morphological changes (Fig 5), indicating kidney injury. Hyperglycemia alone can lead to diabetic nephropathy, a common cause of chronic kidney disease that could lead to end-stage renal disease [36]. Reversal of nephropathy in type-1 diabetes model is not easy achieved, even with tight control of glycemic levels [37]

Superimposition of hypertension and diabetes has been shown to induce accelerated nephropathy in mice [18], and concomitant presence of hypertension and diabetes in humans increases the chances of mice to develop chronic kidney disease [38, 39]. In our study, HFD regimen resulted in protection against kidney injury and impairment in function (Figs 4 and 5). HFD alone has been shown to promote lipotoxicity in the kidney of mice [40], whereas ketogenic diet partially reverts diabetic nephropathy in db/db mice [41]. Taking in consideration the trend to decrease SBP observed in our study, we hypothesize that HFD could lead to cardiovascular protection [35], resulting in kidney protection. Additionally, the metabolic changes induced by HFD could also contribute to kidney protection. Due to the complexity of superimposing hypertension, diabetes, and high fat diet, the mechanisms are not clearly understood. Additional investigation would be necessary to understand the role of lipid-rich diets on CVD and CKD.

Morphological analysis of liver samples showed hepatic damage by the development of significant steatosis, ballooning, and inflammation (Fig 6) in hypertensive diabetic mice fed HFD. Such phenotype recapitulates the NAFLD [42]. High fat diet is known to induce liver damage [42, 43]. Although there is a high prevalence of steatosis among patients with NAFLD induced by HFD, only a small percentage will develop inflammation and fibrosis [42]. Our results show that WT treated with HFD show less ballooning and inflammation than LinSTZ HFD group, reinforcing the importance of comorbidities in aggravation of liver damage.

In summary, we showed that LinSTZ HFD is not a suitable model for MS, but our interesting findings on HFD in kidney function and morphology should be further investigated to unveil the contradictory mechanisms by which a high lipid diet can protect against CKD.

## Supporting information

S1 Table List of primers used for qPCR. (DOCX)

S1 Fig Expression of Renin and Ang II levels are not affected by liver damaged induced by HFD. (TIF)

## References

[1] Polotsky VY. Mouse model of the metabolic syndrome: the quest continues. J Appl Physiol. 2007;102: 2088–2089. doi: 10.1152/japplphysiol.00219.2007 17317878

[2] Wong SK, Chin K-Y, Suhaimi FH, Fairus A, Ima-Nirwana S. Animal models of metabolic syndrome: a review. Nutr Metab (Lond). 2016;13: 65. doi: 10.1186/s12986-016-0123-9 27708685 PMC5050917

[3] Tozawa M, Iseki K, Iseki C, Kinjo K, Ikemiya Y, Takishita S. Blood Pressure Predicts Risk of Developing End-Stage Renal Disease in Men and Women. Hypertension. 2003;41: 1341–1345. doi: 10.1161/01.HYP.0000069699.92349.8C 12707291

[4] Hirayama A, Konta T, Kamei K, Suzuki K, Ichikawa K, Fujimoto S, et al. Blood Pressure, Proteinuria, and Renal Function Decline: Associations in a Large Community-Based Population. Am J Hypertens. 2015;28: 1150–1156. doi: 10.1093/ajh/hpv003 25673040

[5] Tune JD, Goodwill AG, Sassoon DJ, Mather KJ. Cardiovascular consequences of metabolic syndrome. Transl Res. 2017;183: 57–70. doi: 10.1016/j.trsl.2017.01.001 28130064 PMC5393930

[6] Isomaa B, Almgren P, Tuomi T, Forsén B, Lahti K, Nissén M, et al. Cardiovascular Morbidity and Mortality Associated With the Metabolic Syndrome. Diabetes Care. 2001;24: 683–689. doi: 10.2337/diacare.24.4.683 11315831

[7] Tabish SA. Is Diabetes Becoming the Biggest Epidemic of the Twenty-first Century? Int J Health Sci (Qassim). 2007;1: V–VIII. 21475425 PMC3068646

[8] Pi-Sunyer FX. Obesity and Hypertension. Obes Manag. 2009;5: 57–61. doi: 10.1089/obe.2009.0204

[9] Musso G, Gambino R, Tabibian JH, Ekstedt M, Kechagias S, Hamaguchi M, et al. Association of Non-alcoholic Fatty Liver Disease with Chronic Kidney Disease: A Systematic Review and Meta-analysis. Woodward M, editor. PLoS Med. 2014;11: e1001680. doi: 10.1371/journal.pmed.1001680 25050550 PMC4106719

[10] Byrne CD, Targher G. NAFLD: A multisystem disease. J Hepatol. 2015;62: S47–S64. doi: 10.1016/j.jhep.2014.12.012 25920090

[11] Burger D, Reudelhuber TL, Mahajan A, Chibale K, Sturrock ED, Touyz RM. Effects of a domain-selective ACE inhibitor in a mouse model of chronic angiotensin II-dependent hypertension. Clin Sci. 2014;127: 57–63. doi: 10.1042/CS20130808 24506807

[12] Prescott G, Silversides DW, Chiu SML, Reudelhuber TL. Contribution of circulating renin to local synthesis of angiotensin peptides in the heart. Physiol Genomics. 2000;4: 67–73. doi: 10.1152/physiolgenomics.2000.4.1.67 11074015

[13] Graham ML, Janecek JL, Kittredge JA, Hering BJ, Schuurman H-J. The streptozotocin-induced diabetic nude mouse model: differences between animals from different sources. Comp Med. 2011;61: 356–360. 22330251 PMC3155402

[14] Saadane A, Lessieur EM, Du Y, Liu H, Kern TS. Successful induction of diabetes in mice demonstrates no gender difference in development of early diabetic retinopathy. Lewin AS, editor. PLoS ONE. 2020;15: e0238727. doi: 10.1371/journal.pone.0238727 32941450 PMC7498040

[15] Leiter EH. Multiple low-dose streptozotocin-induced hyperglycemia and insulitis in C57BL mice: Influence of inbred background, sex, and thymus. Proc Natl Acad Sci USA. 1982;79: 630–634. doi: 10.1073/pnas.79.2.630 6210909 PMC345800

[16] Licholai JA, Nguyen KP, Fobbs WC, Schuster CJ, Ali MA, Kravitz AV. Why Do Mice Overeat High-Fat Diets? How High-Fat Diet Alters the Regulation of Daily Caloric Intake in Mice: Why Do Mice Overeat High-Fat Diets? Obesity. 2018;26: 1026–1033. doi: 10.1002/oby.22195 29707908 PMC5970071

[17] Wang C-Y, Liao JK. A Mouse Model of Diet-Induced Obesity and Insulin Resistance. In: Weichhart T, editor. mTOR. Totowa, NJ: Humana Press; 2012. pp. 421–433. doi: 10.1007/978-1-61779-430-8_27 PMC3807094 22125082

[18] Thibodeau J-F, Holterman CE, Burger D, Read NC, Reudelhuber TL, Kennedy CRJ. A Novel Mouse Model of Advanced Diabetic Kidney Disease. Abe H, editor. PLoS ONE. 2014;9: e113459. doi: 10.1371/journal.pone.0113459 25514595 PMC4267730

[19] Moore JF, Sharer JD. Methods for Quantitative Creatinine Determination. Curr Protoc Hum Genet. 2017;93. doi: 10.1002/cphg.38 28384398

[20] Furman BL. Streptozotocin‐Induced Diabetic Models in Mice and Rats. Curr Protoc Pharmacol. 2015;70. doi: 10.1002/0471141755.ph0547s70 26331889

[21] Chang A, Kramer H. Effect of Obesity and the Metabolic Syndrome on Incident Kidney Disease and the Progression to Chronic Kidney Failure. Nutritional Management of Renal Disease. Elsevier; 2013. pp. 445–456. doi: 10.1016/B978-0-12-391934-2.00028-X

[22] Andres-Hernando A, Lanaspa MA, Kuwabara M, Orlicky DJ, Cicerchi C, Bales E, et al. Obesity causes renal mitochondrial dysfunction and energy imbalance and accelerates chronic kidney disease in mice. Am J Physiol Renal Physiol. 2019;317: F941–F948. doi: 10.1152/ajprenal.00203.2019 31411075

[23] Nashar K, Egan B. Relationship between chronic kidney disease and metabolic syndrome: current perspectives. Diabetes Metab Syndr Obes. 2014; 421. doi: 10.2147/DMSO.S45183 25258547 PMC4173754

[24] Wu KK, Huan Y. Streptozotocin‐Induced Diabetic Models in Mice and Rats. Curr Protoc Pharmacol. 2008;40. doi: 10.1002/0471141755.ph0547s40 22294227

[25] R N, J Z, V C, Jf T, Kd B, Rl H. Collecting duct PGE2 responses reduce water loss with empagliflozin in mice with type 2 diabetes mellitus. J Clini Nephrol. 2021;5: 023–030. doi: 10.29328/journal.jcn.1001069

[26] Caramori ML, Parks A, Mauer M. Renal Lesions Predict Progression of Diabetic Nephropathy in Type 1 Diabetes. J Am Soc Nephrol. 2013;24: 1175–1181. doi: 10.1681/ASN.2012070739 23687360 PMC3699823

[27] Carvalho AL, DeMambro VE, Guntur AR, Le P, Nagano K, Baron R, et al. High fat diet attenuates hyperglycemia, body composition changes, and bone loss in male streptozotocin‐induced type 1 diabetic mice. J Cell Physiol. 2018;233: 1585–1600. doi: 10.1002/jcp.26062 28631813 PMC5673543

[28] Azushima K, Gurley SB, Coffman TM. Modelling diabetic nephropathy in mice. Nat Rev Nephrol. 2018;14: 48–56. doi: 10.1038/nrneph.2017.142 29062142

[29] Skovsø S. Modeling type 2 diabetes in rats using high fat diet and streptozotocin. J Diabetes Invest. 2014;5: 349–358. doi: 10.1111/jdi.12235 25411593 PMC4210077

[30] Postic C, Dentin R, Denechaud P-D, Girard J. ChREBP, a Transcriptional Regulator of Glucose and Lipid Metabolism. Annu Rev Nutr. 2007;27: 179–192. doi: 10.1146/annurev.nutr.27.061406.093618 17428181

[31] Ipsen DH, Lykkesfeldt J, Tveden-Nyborg P. Molecular mechanisms of hepatic lipid accumulation in non-alcoholic fatty liver disease. Cell Mol Life Sci. 2018;75: 3313–3327. doi: 10.1007/s00018-018-2860-6 29936596 PMC6105174

[32] Gorriz JL, Martinez-Castelao A. Proteinuria: detection and role in native renal disease progression. Transplant Rev. 2012;26: 3–13. doi: 10.1016/j.trre.2011.10.002 22137726

[33] Saratlija Novakovic Z, Glavina Durdov M, Puljak L, Saraga M, Ljutic D, Filipovic T, et al. The interstitial expression of alpha-smooth muscle actin in glomerulonephritis is associated with renal function. Med Sci Monit. 2012;18: CR235–CR240. doi: 10.12659/msm.882623 22460095 PMC3560831

[34] Kramann R, Menzel S. of Kidney Fibrosis. In: Hinz B, Lagares D, editors. Myofibroblasts. New York, NY: Springer US; 2021. pp. 323–338. doi: 10.1007/978-1-0716-1382-5_22 34028752

[35] Pour Abbasi MS, Shojaei N, Farhangi MA. Low‐carbohydrate diet score is associated with improved blood pressure and cardio‐metabolic risk factors among obese adults. Physiol Rep. 2022;10. doi: 10.14814/phy2.15375 35822420 PMC9277405

[36] Bikbov B, Purcell CA, Levey AS, Smith M, Abdoli A, Abebe M, et al. Global, regional, and national burden of chronic kidney disease, 1990–2017: a systematic analysis for the Global Burden of Disease Study 2017. The Lancet. 2020;395: 709–733. doi: 10.1016/S0140-6736(20)30045-3 32061315 PMC7049905

[37] Kowluru RA, Abbas SN, Odenbach S. Reversal of hyperglycemia and diabetic nephropathy. J Diabetes Complications. 2004;18: 282–288. doi: 10.1016/j.jdiacomp.2004.03.002 15337502

[38] Shi W, Wang H, Zhou Y, Sun Y, Chen Y. Synergistic interaction of hypertension and diabetes on chronic kidney disease: Insights from the National Health and Nutrition Examination Survey 1999–2006. J Diabetes Complications. 2020;34: 107447. doi: 10.1016/j.jdiacomp.2019.107447 31818688

[39] Wang J, Lv J, He K, Wang F, Gao B, Zhao M, et al. Association of left ventricular hypertrophy and functional impairment with cardiovascular outcomes and mortality among patients with chronic kidney disease, results from the C‐STRIDE study. Nephrology. 2022;27: 327–336. doi: 10.1111/nep.14009 34843156

[40] Yamamoto T, Takabatake Y, Takahashi A, Kimura T, Namba T, Matsuda J, et al. High-Fat Diet–Induced Lysosomal Dysfunction and Impaired Autophagic Flux Contribute to Lipotoxicity in the Kidney. JASN. 2017;28: 1534–1551. doi: 10.1681/ASN.2016070731 27932476 PMC5407727

[41] Poplawski MM, Mastaitis JW, Isoda F, Grosjean F, Zheng F, Mobbs CV. Reversal of Diabetic Nephropathy by a Ketogenic Diet. Stadler K, editor. PLoS ONE. 2011;6: e18604. doi: 10.1371/journal.pone.0018604 21533091 PMC3080383

[42] Leoni S, Tovoli F, Napoli L, Serio I, Ferri S, Bolondi L. Current guidelines for the management of non-alcoholic fatty liver disease: A systematic review with comparative analysis. World J Gastroenterol. 2018;24: 3361–3373. doi: 10.3748/wjg.v24.i30.3361 30122876 PMC6092580

[43] Recena Aydos L, Aparecida do Amaral L, Serafim de Souza R, Jacobowski AC, Freitas dos Santos E, Rodrigues Macedo ML. Nonalcoholic Fatty Liver Disease Induced by High-Fat Diet in C57bl/6 Models. Nutrients. 2019;11: 3067. doi: 10.3390/nu11123067 31888190 PMC6949901

