# Supplementary material for: High fat diet is protective against kidney injury in hypertensive-diabetic mice, but leads to liver injury
Source: PLoS One. 2023 Feb 2;18(2):e0281123. doi: 10.1371/journal.pone.0281123 (PMC9894391; doi:10.1371/journal.pone.0281123)
Supplement: S1 Table — (DOCX) [file pone.0281123.s001.docx]

| Product name | Size (bp) | Primer sequences (DNA) | Tm value (℃) |
| --- | --- | --- | --- |
| human Renin | 161 | **Sense:** 5’ -AAGTGCAGCCGTCTCTACAC- 3’ | 58 |
| (hRen) |  | **Anti-sense:** 5’ -GTGATTCCACCCACGGTGAT- 3’ |  |
| mouse Renin | 184 | **Sense:** 5’ -GCACCGCTACCTTTGAACGA- 3’ | 58 |
| (mRen) |  | **Anti-sense:** 5’ -CCGTAGTACTGGGTATTCAGGT- 3’ |  |
| mouse GAPDH | 151 | **Sense:** 5’ -ACTCCACTCACGGCAAATTC- 3’ | 58 |
|  |  | **Anti-sense:** 5’ -TCTCCATGGTGGTGAAGACA- 3’ |  |
| mouse 18S | 61 | **Sense:** 5’ -ATGGTAGTCGCCGTGCCTAC- 3’ | 60 |
|  |  | **Anti-sense:** 5’ -CCGGAATCGAACCCTGATT- 3’ |  |
| Abbreviations: base pairs (bp) and melting temperature (Tm) | | | |

**Table S1**. List of primers
