# Supplementary figures and images for: High fat diet is protective against kidney injury in hypertensive-diabetic mice, but leads to liver injury
Source: PLoS One. 2023 Feb 2;18(2):e0281123. doi: 10.1371/journal.pone.0281123 (PMC9894391; doi:10.1371/journal.pone.0281123)

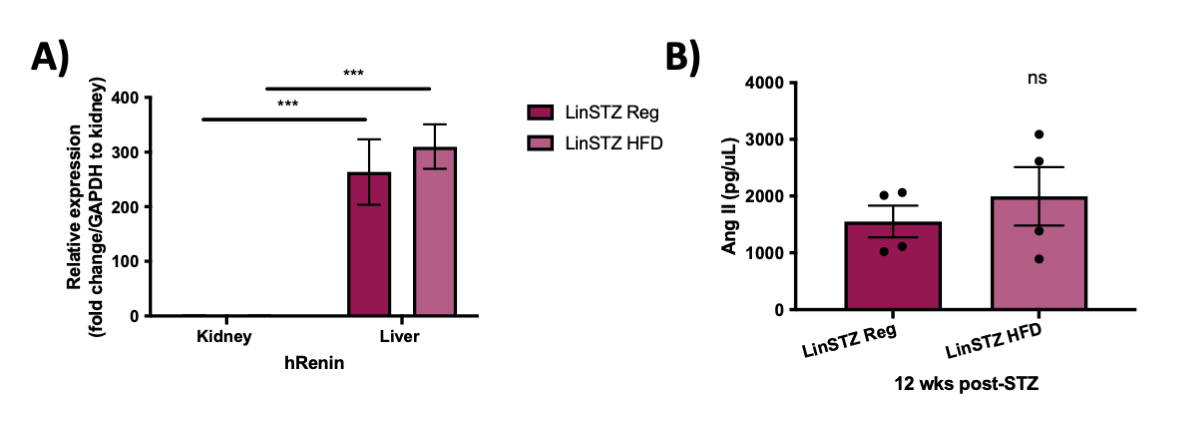

Supplement: S1 Fig — (TIF) [file pone.0281123.s002.tif]
